# A preliminary survey of filarial parasites in dogs and cats in Sri Lanka

**DOI:** 10.1371/journal.pone.0206633

**Published:** 2018-11-02

**Authors:** Chandana H. Mallawarachchi, Nilmini T. G. A. Chandrasena, Susiji Wickramasinghe, Ranjan Premaratna, Nilmini Y. I. S. Gunawardane, Navoda S. M. S. M. Mallawarachchi, Nilanthi R. de Silva

**Affiliations:** 1 Postgraduate Institute of Medicine, University of Colombo, Colombo, Sri Lanka; 2 Department of Parasitology, Faculty of Medicine, University of Kelaniya, Kelaniya, Sri Lanka; 3 Department of Parasitology, Faculty of Medicine, University of Peradeniya, Peradeniya, Sri Lanka; 4 Department of Medicine, Faculty of Medicine, University of Kelaniya, Kelaniya, Sri Lanka; 5 Molecular Medicine Unit, Faculty of Medicine, University of Kelaniya, Kelaniya, Sri Lanka; Sciensano, BELGIUM

## Abstract

Human brugian filariasis has re-emerged in Sri Lanka after a quiescent period of four decades. This study investigated the prevalence of canine and feline filarial parasites in three localities with human sub-periodic brugian filariasis, in order to determine their potential reservoir status. All reachable dogs and cats, both stray and domestic, within a 350m radius of an index case of brugian filariasis in three locations (Madampe, Wattala and Weliweriya) were screened for microfilariae using Giemsa stained thick blood smears. A representative sample of canine and feline blood samples positive for *Brugia* spp. microfilariae by microscopy, from each of the three locations, were further analyzed by PCR with specific primers for internal transcribed spacer region 2 (ITS2) of the ribosomal DNA. A total of 250 dogs and 134 cats were screened. The overall microfilaraemia rates were high among both dogs (68.8%) and cats (47.8%). The prevalence of microfilaraemia was significantly higher among dogs than cats (p<0.05). Two filarial species were identified based on morphology of microfilariae: *Dirofilaria (Nochtiella) repens* (dogs, 54.4% and cats, 34.3%) and *Brugia* spp. (dogs, 51.6% and cats, 30.6%). PCR analysis of canine (n = 53) and feline (n = 24) samples elicited bands in the region of 615bp, which confirmed *Brugia malayi* infection. Co-infection with *D*.*(N*.*) repens* was detected by PCR with an additional band at 484bp, in 36 canine and 17 feline samples. Overall microfilaraemia rates of dogs (81.8%) and cats (75%) in Madampe (rural) were significantly higher than in urbanized Wattala (dogs, 62.4% and cats, 26.0%) (p<0.05). High rates of zoonotic filarial infections strongly implicate dogs and cats as potential reservoirs for human dirofilariasis and brugian filariasis in Sri Lanka.

## Background

Human brugian filariasis has re-emerged in Sri Lanka after a quiescent period of four decades [1]. In contrast to the periodic brugian filariasis that was previously endemic, the re-emergent strain is subperiodic, suggestive of a zoonotic origin [2]. Sri Lanka is also known for its high case burden of dirofilariasis caused by the zoonotic filarial parasite, *D*. *(N) repens* [3–7]. Cats and dogs are well-recognized reservoirs for *B*.*malayi* and *D*. *(N) repens* [8–12].

Canine filariases have been previously reported from Sri Lanka [3, 13]. The filarial parasites reported were *D*.*(N) repens* and *Brugia ceylonensis* from the Western province [13], *B*.*malayi* and *D*.*(N) repens* from Western and North Central provinces (unpublished data). Since these zoonotic parasites have the potential to cause disease in humans, their prevalence is of veterinary and public health significance [8–10].

The adult worms of the zoonotic strain of subperiodic *B*. *malayi* inhabit the lymphatic system of the human hosts and produce microfilariae which circulate in the peripheral blood. Clinical manifestations of infection are similar to those of human lymphatic filarial species [14]. Lymphatic filarial disease caused by subperiodic *B*.*malayi* in the Southeast Asian region is considered zoonotic due to existence of animal reservoir hosts such as dogs and cats [15, 16]. Little is known regarding canine brugian filariases in Sri Lanka [13] and information on feline filarids is non-existent.

*Dirofilaria* species in Sri Lanka belong to the subgenus *Nochtiella*, and are represented by *D*. *(N*.*) repens*. These parasites are invariably in the subcutaneous tissues of their natural hosts, dogs and cats [3, 14]. In human infections the parasites rarely mature sufficiently to produce microfilariae; thus humans are dead end hosts. Clinical manifestations include subcutaneous nodules, ocular lesions and even meningo-encephalitis, and infections frequently require surgical intervention for removal of the worms [3–8, 14, 17].

Prevalence rates of canine dirofilariasis of 30–60% have been reported from Sri Lanka in the past [3, 13]. Feline filariases in Sri Lanka have not been reported. This study aimed to detect and differentiate zoonotic filarial parasites among dogs and cats inhabiting three localities in Sri Lanka in which human brugian filariasis has recently re-emerged, in order to assess their potential as a source of infection.

## Methods

The surveys were done from December 2016—June 2017. Site selection was based on the occurrence of human brugian infections [1, 2]. A single case each was reported from the three localities, Wattala, Weliweriya and Madampe (S1 Fig). The GPS coordinates of the households of these index cases were obtained at site visits using a handheld GPS monitor (Montana 610 from Garmin Europe) and mapped using ArcGIS 10. Surveillance sites spanning a radius of 350m (mean flight range of vector *Mansonia* spp.) were demarcated around the households of index cases.

Both Wattala and Weliweriya are in the district of Gampaha, in the Western Province of Sri Lanka. Wattala (7.0025° N, 79.9099° E), is a coastal suburb situated close to the commercial capital, Colombo, while Weliweriya (7.0319° N, 80.0283° E) is less urbanized and situated towards the interior of the country. Madampe (7.4972°N, 79.8333° E) in the district of Puttalam, is in the North Western Province and is the least urbanized of the three sites. All three sites have a warm wet climate with ambient temperatures between 22° and 37°C.

All reachable cats and dogs (stray and domestic) within the surveillance sites were screened for microfilariae using thick blood smears (TBS) prepared from blood collected by an ear-lobe prick. The dogs were physically restrained by their owners, while more aggressive and stray dogs were sedated with a dose of 1-3mg/kg bodyweight of xylazine prior to bleeding. The cats were held tightly by their owners while more troublesome ones were held in cages and an ear lobe was extracted for bleeding. Bleeding was done by a trained veterinarian. The earlobe area was shaved and cleaned with antiseptics. Capillary blood was obtained by pricking the edge of the ear with a sterile 23 gauge needle. A sample of blood from each animal was spotted on to filter paper and stored for molecular analysis. Two sets of TBS were prepared from each animal, stained with Giemsa and read by two independent experts at the Departments of Parasitology, Faculties of Medicine, Universities of Kelaniya and Peradeniya, Sri Lanka. The microfilariae were identified on the basis of the following morphological features: presence or absence of sheath, total body length, the presence and measurements of cephalic space and appearance of the tail (straight or curved, blunt appearance and the presence or absence of nuclei) [18, 19, 20, 21].

A representative sample of *Brugia* spp. microfilariae positive blood of dogs and cats were randomly selected from the three surveillance sites for polymerase chain reaction (PCR). Parasite DNA was extracted from filter papers using the ReliaPrep Blood DNA Miniprep System (Promega Corporation, United States of America, Catalog number A5082) according to manufacturer’s instructions. The method described by Rishniw et. al. to discriminate between six species of canine microfilariae with a single polymerase chain reaction using pan-filarial primers (DIDR-F1 5'-AGT GCG AAT TGC AGA CGC ATT GAG-3' and DIDR-R1 5'-AGC GGG TAA TCA CGA CTG AGT TGA-3') that span the internal transcribed spacer region 2 (ITS2) of the ribosomal DNA, was employed to amplify the target DNA region [22]. Specimens of DNA of *B*.*malayi* (Liverpool School of Tropical Medicine, Liverpool, UK) and *D*. *(N*.*) repens* (adult specimen from Department of Parasitology, University of Kelaniya) served as positive controls and sterile distilled water served as a negative control. The products were visualized by electrophoresis on a 2% agarose gel. Species identification was based on the molecular weights of the amplicons [22].

### Analysis

Data was entered into a MS-Excel spread sheet and exported into SPSS for Windows (Ver.22) for analysis. Frequencies and proportions were calculated. Two sample Z test was applied for comparison of proportions. P values less than 0.05 were considered as statistically significant.

### Ethics

Ethical clearance for the study was obtained from the Ethics Review Committees of the Faculty of Medicine, University of Kelaniya (P/108/09/2016) and the Medical Research Institute, Colombo (40/2016). Approval was obtained from relevant Provincial Directors (Western and Northwestern) of animal production and health and divisional veterinary officers. Informed written consent was obtained from the owners of domestic cats and dogs.

## Results

A total of 250 dogs (stray 10, domestic 240) and 134 cats (stray 03, domestic 131) were screened at the three surveillance sites. Male: female ratios of dogs and cats screened were 2.2: 1 and 1.5:1 respectively. The mean age of the dogs and cats were 3.6 (SD 2.99) and 3.05 (SD 2.75) years respectively.

**Fig 1:**
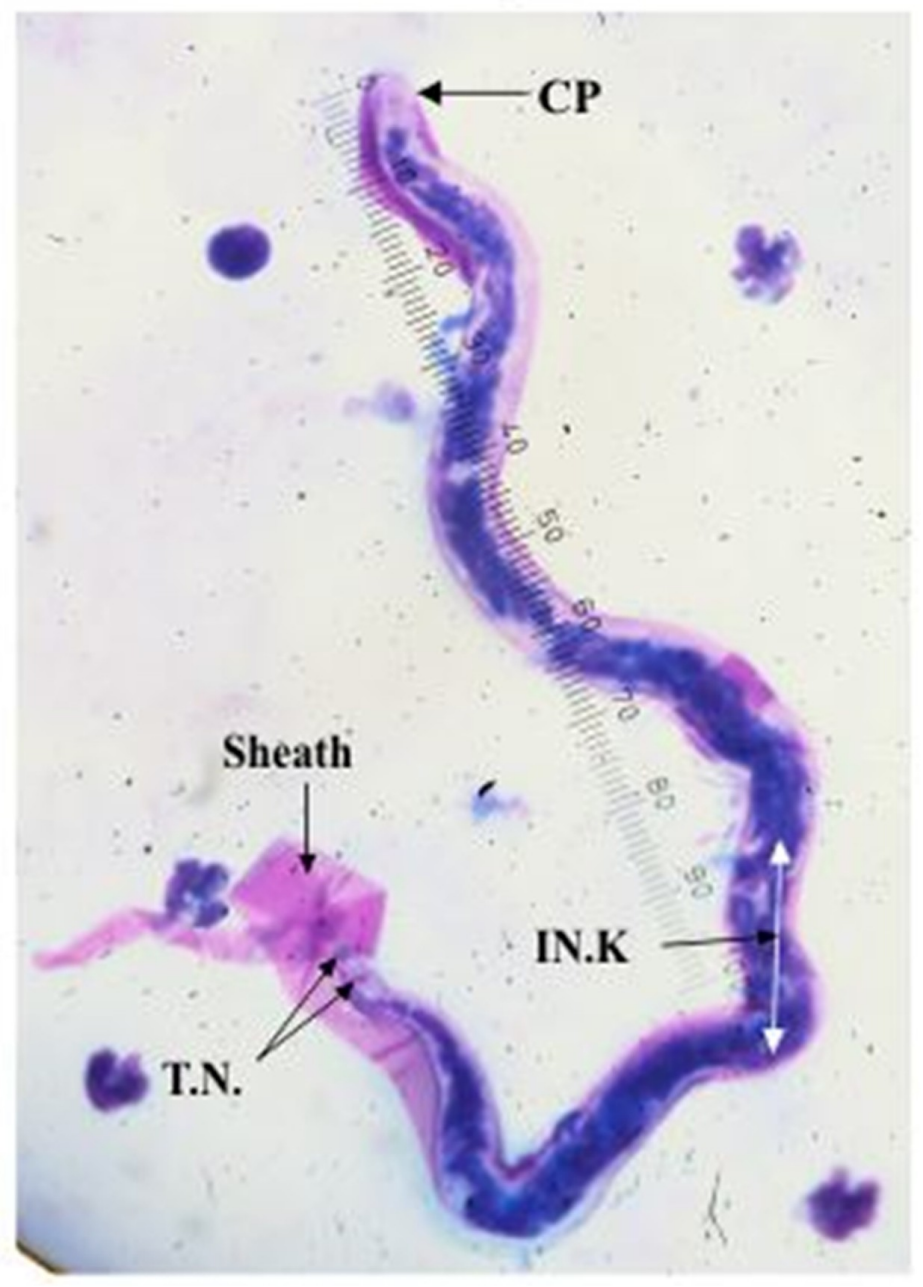
Microfilaria of *B*. *malayi*. CP- Cephalic space IN.K.- Innen-korper T.N.- Terminal nuclei.

**Fig 2:**
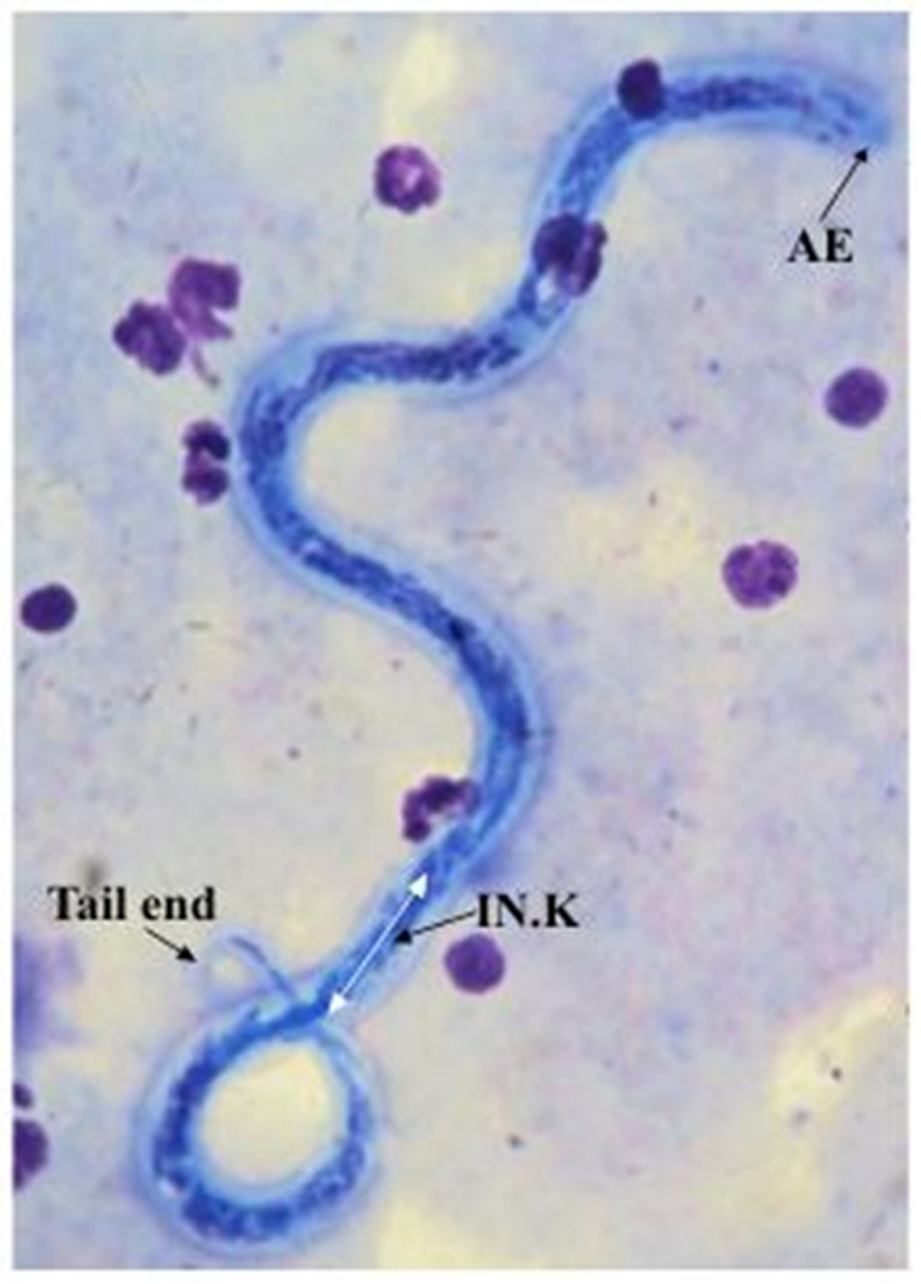
Microfilaria of *D*.*(N*.*) repens*. AE—Anterior end with two distinct nuclei IN.K.—Innen- korper.

Two species of filarial parasites were identified based on previously published morphological characteristics as given in Table 1. Sheathed microfilariae with a mean body length of 226 μm (SD 18.5), a mean cephalic space length of 5.6μm (SD 0.87) and a pair of tail nuclei, were identified as belonging to genus *Brugia* [18, 19, 20] as shown in Fig 1. Unsheathed microfilarae with a mean body length of 293 μm (SD 17.5) and a pair of cephalic nuclei were identified as *D*.*(N*.*) repen*s [21] as shown in Fig 2.

**Table 1:** Measurements of *B*.*malayi* microfilaria from the current survey compared with *B*.*malayi*, *B*.*pahangi* and *B*.*ceylonensis* from scientific literature ^a^.

MF measurements	*B*.*malayi* MF current study		*B*.*malayi* MF	*B*.*pahangi*MF	*B*.*ceylonensis*MF
	dogs	cats			
Mean total body length (μm)	225.82	226.31	220–221	245.8	220–275
Mean length of Innenkorper (μm)	38.04	38.46	31.1–32.29	49.5	NA
Mean length of cephalic space (μm)	5.65	5.47	NA	NA	6.3–6.7
Mean width of cephalic space (μm)	4.87	4.84	NA	NA	NA
Cephalic space width: length ratio	0.86	0.88	0.78	NA	NA

^a^ Data obtained from [13, 18, 19, 20, 21].

NA- not available.

MF- microfilariae.

**Fig 3:**
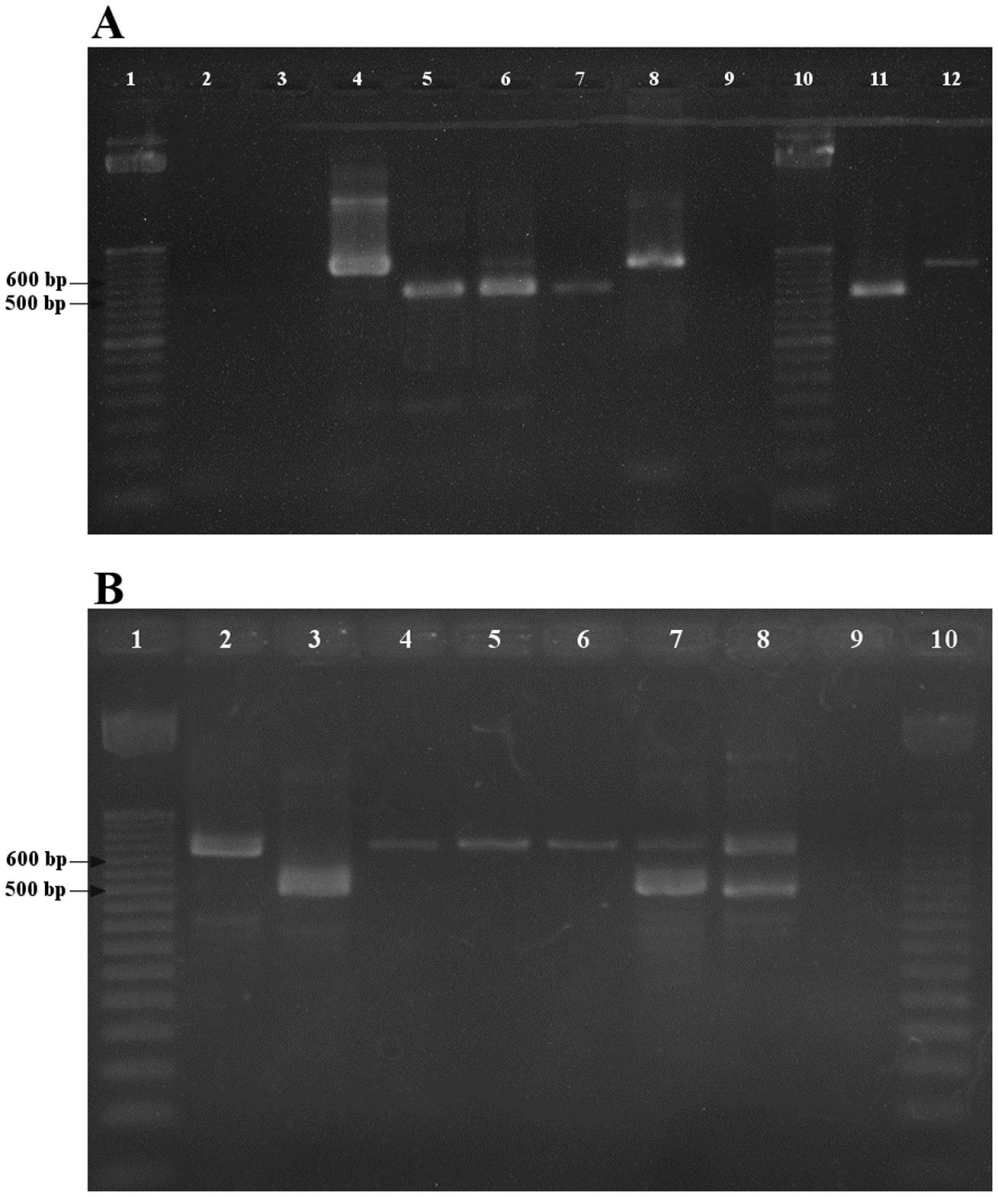
Gel electrophoresis of PCR products following amplification with panfilarial primers. **A**. Lanes 1 and 10–50 bp DNA marker, 2- Dog DNA, 3- Cat DNA, 4 –*B*. *malayi* positive control, 5 –*D*. *repens* positive control, 6—Co-infection *B*.*malayi* and *D*.*repens* 7- Mono-infection *D*.*repens*, 8 –Mono-infection *B*.*malayi* (6 to 8 Dog, Madampe), 9- Negative control, 11—Mono-infection *D*.*repens* and 12—Mono-infection *B*.*malayi* (11 and 12 Cat, Madampe). **B**. Lanes 1 and 10–50 bp DNA marker, 2 and 4—Mono-infection *B*.*malayi*, 3—Mono-infection *D*.*repens* (2 to 4 Dog, Wattala), 5 and 6 –Mono-infection *B*.*malayi* (Cat, Wattala), 7 and 8 –Co-infection *B*.*malayi* and *D*.*repens* dog and cat respectively (Weliweriya), 9- Negative control.

PCR analyses carried out with panfilarial primers on canine (n = 54) and feline (n = 25) blood samples positive for *Brugia* spp. MF elicited the expected bands in the region of 615bp which confirmed *B*. *malayi* infection as shown in Fig 3. Co-infection with *D*. *(N*.*) repens* was detected by an additional band at 484bp [22] in 36 canine and 17 feline (Fig 3).

The overall rates of microfilariaemia were high among both dogs (172/250, 68.8%) and cats (64/134, 47.8%). Dogs had a significantly higher rate of microfilaraemia than cats (p<0.05), as shown in Table 2. Of the two filarial species, *D*.*(N*.*) repen*s was more common among both dogs and cats. The prevalence of microfilaremia among domestic dogs and cats was 69.2% (166/240) and 48.1% (63/131), respectively.

**Table 2:** Prevalence of microfilaraemia among dogs and cats based on Giemsa stained thick blood smears.

	Sample size	*B*.*malayi* mono-infections N (%)	*D*.*(N*.*) repens* mono-infections N (%)	Co-infections N (%)	Total infected N (%)
Dogs	250	36 (14.4)	43 (17.2)	93 (37.2)	172 (68.8)
Cats	134	18 (13.4)	23 (17.2)	23 (17.2)	64 (47.8)

The overall prevalence of microfilaraemia among dogs and cats was significantly higher in Madampe compared to Wattala and Weliweriya (p < 0.01), as shown in Table 3.

**Table 3:** Comparison of area-wise prevalence of microfilaraemia among cats and dogs.

Animal participant	Area	Total screened	Total infected	Infected (%)	p-value
**Dogs**	Madampe	77	63	81.82%	
	Wattala	109	68	62.39%	^a^0.004
	Weliweriya	64	41	64.06%	^b^0.016
	Total	250	172	68.80%	^c^0.8
**Cats**	Madampe	52	39	75%	
	Wattala	50	13	26%	^a^0.0001
	Weliweriya	32	12	37.50%	^b^0.0006
	Total	134	64	47.76%	^c^0.27

^a^ Comparison of infection rates between Madampe and Wattala.

^b^ Comparison of infection rates between Madampe and Weliweriya.

^c^ Comparison of infection rates between Wattala and Weliweriya.

Similar results were obtained when the prevalence of individual zoonotic filarial species (*B*. *malayi* and *D*. *(N*.*) repens*) in the three sites were compared. Madampe had significantly higher rates of *B*. *malayi* infections (dogs 68.8%, n = 53 and cats 55.8%, n = 29) compared to Wattala (dogs 49.5%, n = 54 and cats 20%, n = 10) and Weliweriya (dogs 34.4%, n = 22 and cats 6.3%, n = 2) (p < 0.01). Similarly, rates of *D*. *(N*.*) repens* infections among dogs and cats was also significantly higher in Madampe (dogs 63.6%, n = 49 and cats 51.9%, n = 27) compared to Wattala (dogs 46.8%, n = 51 and cats 16%, n = 8) and Weliweriya (dogs 56.3%, n = 36 and cats 34.4%, n = 11) (p < 0.05).

## Discussion

This is the first report of a simultaneous survey of both cats and dogs for filarial parasites in Sri Lanka. Microscopic examination of Giemsa stained blood smears detected rates of microfilaraemia that were much higher than those reported in the past: 45% [13] and 30–60% [3]. It is likely that the actual rates of infections are even higher than this, since the screening tool is known to be of lower sensitivity than the recommended modified Knotts concentration technique [23]. Nevertheless, the present findings provide an indication of the gravity of the situation from a veterinary and public health perspective.

Of the two species of filarial parasites detected, *D*.*(N*.*)repens* had a slightly higher overall prevalence among both animals than *B*.*malayi*, but in Madampe, a more rural area, *B*.*malayi* was commoner. The overall rates of canine and feline filarial infections observed in the current survey (68.8% and 47.8% in dogs and cats respectively) are much higher than those reported from other countries in the region. Filarial parasites among cats have been mostly studied in Southeast Asia where cats were implicated as reservoir hosts for human lymphatic filariasis [11, 15, 16, 18, 20 24]. The prevalence of microfilaraemia among cats in Malaysia ranged from 20.6% (*B*.*malayi*) in the Peninsular State [16] to 23.5% (*B*.*pahangi*) in Selangor state [11]; while the rates in Southern Thailand and Indonesia were 28.3% (*B*.*malayi*) [18] and 18.8% (*B*.*pahangi*) [24] respectively.

The high prevalence of microfilaraemia among dogs in the current study highlights their importance as potential reservoir hosts for zoonotic filariae. The state of Kerala has reported higher rates of canine microfilaraemia (80%) among a population of symptomatic canines [25] but the rates of infection among domestic canines in the Alappuzha district of Kerala was relatively lower (42.68%), which could be a reflection of the veterinary care received by the study population [26]. A canine survey carried out in four climatic zones in India, ranging from cold montane to wet tropics, reported an even lower prevalence (26.5% overall) [12] probably reflecting the influence of climatic factors on sustenance of the life cycle of filariae. The filariae detected in the Indian survey were *Acanthocheilonema reconditum* and *D*. *(N*.*) repens* whereas in the state of Kerala, 20% of those detected were sheathed microfilaria, presumed to be *B*. *malayi* [12, 25].

Differentiating the sheathed microfilariae of *B*.*malayi* from the non-sheathed microfilariae of *D*.*(N*.*) repens* by microscopy was relatively easy, but species differentiation of *Brugia* microfilariae based on morphology is difficult, as documented by others [14, 26, 27]. PCR with panfilarial primers specific for the ITS2 region of filariae confirmed the identity of the sheathed microfilariae as *B*. *malayi* and the non-sheathed microfilariae as *D*. *(N*.*) repens*.

Comparison of the morphometry of the *B*. *malayi* microfilariae detected in the current survey with those recorded in scientific literature showed similarity in its body length measurements, but the longer innenkorper was more in keeping with *B*. *pahangi* [11]. The width to length ratio of the cephalic space was also less than the 1:2 ratio defined for *B*. *malayi* (Table 1). The morphological variations observed among the *B*. *malayi* microfilariae identified in the current survey could perhaps be a reflection of its zoonotic origin.

Sri Lanka was endemic for both bancroftian and brugian filariasis in the past [28]. Extensive vector control activities resulted in complete clearance of brugian filariasis in the 1960s, and zero cases were reported in an islandwide survey carried out in 1969 [28]. Re-emergence of human brugian filariasis after four decades has raised concerns as it could jeopardize the lymphatic filariasis free status earned by the country [29].

Over 132 cases of subcutaneous and ocular dirofilariasis caused by *D*. *(N*.*) repens* have been documented in Sri Lanka up to year 2000 [4]. Cases continue to occur and a series of seven and two intra-oral infections were reported in 2003 [5] and 2015 [6] respectively. Thirty cases of ocular dirofilariasis were reported over a period of eight years (2006–2014) from a single institution [7]. The emergence of human dirofilariasis as a public health concern appears to be a global phenomenon according to recent reports [30, 31, 32].

Both *B*. *malayi* and *D*.*(N*.*) repens* are associated with substantial morbidity in humans [3–7, 16, 17, 18, 20]. The high prevalence of canine and feline filarial infections is indicative of the extent of the health risk and the importance of treating the animal reservoir. Since chemoprevention with veterinary antifilarial agents (ivermectin) is the mainstay of control, it is vital to disseminate this knowledge to veterinary health authorities for implementation of control measures and thereby reduce the morbidity associated with human dirofilariasis and brugian filariasis in Sri Lanka. Implementation of veterinary control measures is not only of local relevance but may be required on a global scale in regions with high incidence of *D*.*(N*.*)repens*.

The primary objective of this survey was to study the prevalence of zoonotic filariae among domestic animals in the context of the recent re-emergence of human brugian filariasis and the rising trend in ocular and subcutaneous dirofilariasis in Sri Lanka. The results of this survey have provided ample evidence of the role of domestic animals as potential reservoirs of both brugian filariasis and dirofilariasis. It is also evident that establishment of veterinary control programs at this point is essential in order to reduce human exposure to infection.

This preliminary study was carried out among dogs and cats in three small localities (350m radius area of an index human case with brugian filariasis) to identify potential zoonotic reservoir hosts. Thus the rates of canine and feline filariases observed in the current study cannot be generalized to the whole country. Furthermore, the prevalence rates derived are likely to be an underestimate of the true prevalence due to the relatively low sensitivity of the screening tool (TBS). A countrywide survey with more sensitive screening tools could provide more accurate estimation of the prevalence of canine and feline filariases in Sri Lanka. However the primary objective of the survey, i.e. ascertaining the potential reservoir status of domestic animals for the re-emergent human brugian filariasis in Sri Lanka was achieved.

Sri Lanka was certified to have eliminated lymphatic filariasis (bancroftian) as a public health problem in 2016 [29]. The re-emergence of brugian filariasis after four decades may endanger the lymphatic filariasis-free status of the country unless remedial measures are promptly instituted. The One Health approach, with collaboration between diverse disciplines, is recommended to address the complex issues associated with control of zoonoses [33].

## Supporting information

S1 Fig Area map showing the three surveillance sites, Madampe, Weliweriya and Wattala. (TIF)
