# Supplementary figures and images for: A preliminary survey of filarial parasites in dogs and cats in Sri Lanka
Source: PLoS One. 2018 Nov 2;13(11):e0206633. doi: 10.1371/journal.pone.0206633 (PMC6214534; doi:10.1371/journal.pone.0206633)

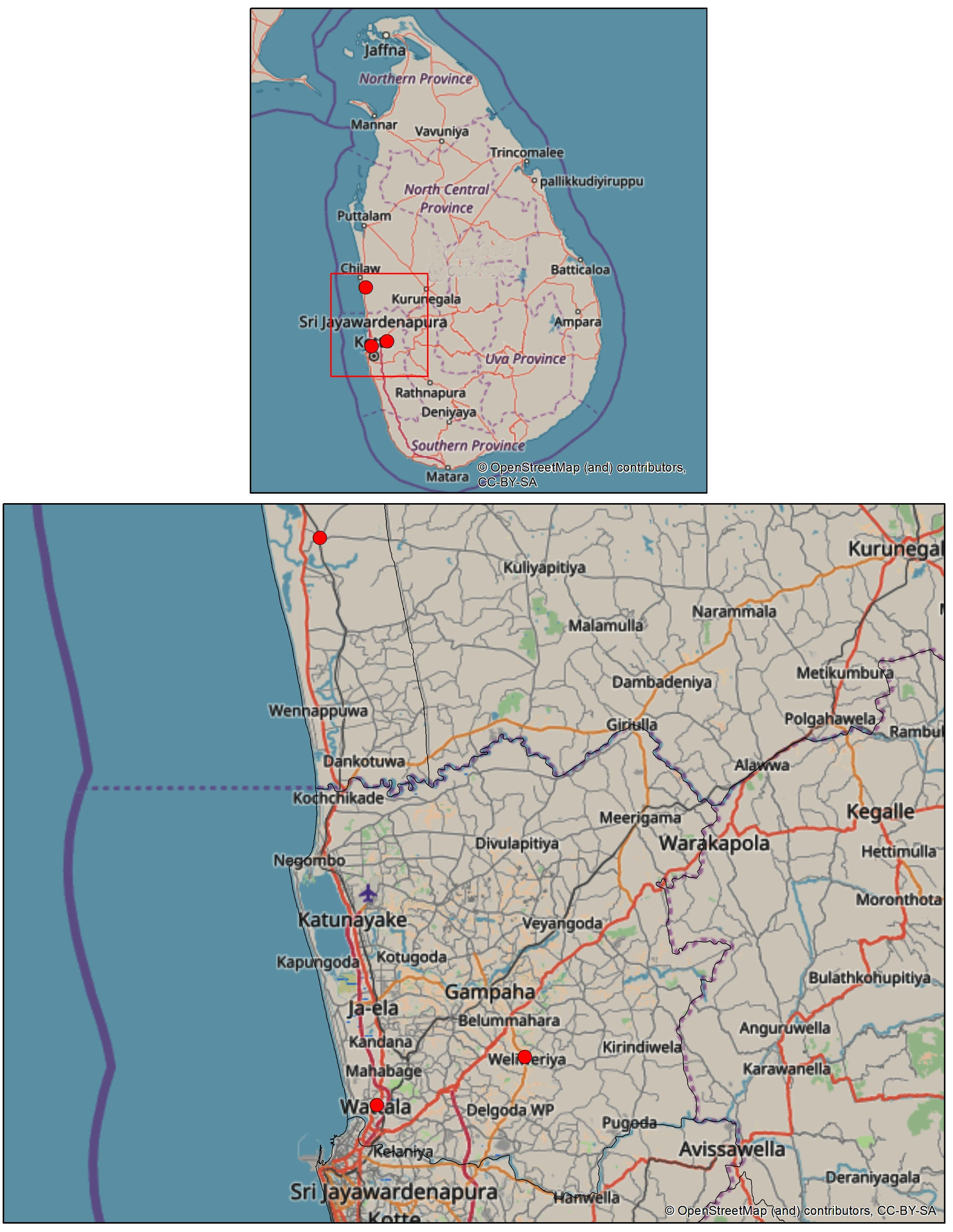

Supplement: S1 Fig — (TIF) [file pone.0206633.s001.tif]
